# Integrated renal histopathology, RNA decay, and protein degradation signatures enhance post-mortem interval prediction using machine-learning models in a veterinary forensic rat model

**DOI:** 10.14202/vetworld.2026.3432-3442

**Published:** 2026-08-08

**Authors:** Om Prakash Choudhary, Albiruni Haryo, Fidi Nur Aini Eka Puji Dameanti

**Affiliations:** 1 Department of Veterinary Anatomy, College of Veterinary Science, Guru Angad Dev Veterinary and Animal Sciences University, Rampura Phul-151103, Punjab, India.; 2 Laboratory of Anatomic Pathology, Faculty of Veterinary Medicine, Universitas Brawijaya, Malang 65151, Indonesia.; 3 Laboratory of Microbiology, Faculty of Veterinary Medicine, Universitas Brawijaya, Malang 65151, Indonesia.

**Keywords:** machine-learning, post-mortem interval, protein degradation, renal autolysis, RNA decay, veterinary forensics, Western blotting, Wistar rat

## Abstract

**Background and Aim:**

Accurate estimation of post-mortem interval (PMI) is essential in veterinary forensics for reconstructing events surrounding death and supporting legal, welfare, and biosecurity investigations. Traditional methods relying on gross and histological changes are often subjective and highly influenced by environmental factors. This study aimed to develop a multimodal molecular approach by integrating renal histopathological autolysis scoring, RNA decay profiles, and protein degradation kinetics, then comparing classical regression with machine-learning models for PMI prediction in a controlled rat model.

**Materials and Methods:**

Kidneys were collected from adult male Wistar rats at 0, 6, 12, 24, and 48 h post-mortem under controlled 25°C conditions. Histopathological autolysis was scored on a 5-point ordinal scale. RNA degradation was assessed by reverse transcription quantitative polymerase chain reaction (RT-qPCR) for heat shock protein 70 (*HSP70*), glyceraldehyde-3-phosphate dehydrogenase (*GAPDH)*, and *18S rRNA*. Protein degradation was evaluated by Western blotting for *HSP70*, Caspase-3, and LC3. Integrated datasets were used to build and compare multiple linear regression, random forest, and support vector regression models using leave-one-out cross-validation.

**Results:**

Autolysis scores increased progressively from 1.2 ± 0.2 at 0 h to 4.7 ± 0.5 at 48 h. RT-qPCR cycle threshold values increased steadily for all RNA targets. Protein markers exhibited distinct kinetics: *HSP70* declined continuously, Caspase-3 peaked at 12 h, and LC3-II plateaued after 24 h. Support vector regression achieved the best performance (mean absolute error of 0.75 h, root mean square error of 1.40 h, R² of 0.99, and 92% of predictions within ±3 h), outperforming random forest and linear regression.

**Conclusion:**

Integrating renal histopathology, RNA decay, and protein degradation signatures with machine-learning models, particularly support vector regression, enables accurate PMI prediction under controlled conditions. This multimodal framework offers a promising template for advancing molecular veterinary forensic science, though validation under variable field conditions is required.

## INTRODUCTION

Post-mortem interval (PMI) estimation is fundamental in forensic investigations because it supports reconstruction of events surrounding death and strengthens interpretation of lesions and environmental findings [1]. Although human forensic science has developed physical, biochemical, and entomological approaches, early-to-intermediate PMI estimation remains challenging and context-dependent [2, 3]. In veterinary forensics, uncertainty is amplified by species diversity, variation in body size and carcass handling, and frequent extrapolation of human-based methods without validation in animal models [4]. As animal deaths increasingly intersect with legal, welfare, biosecurity, and insurance contexts, the need for robust, species-appropriate PMI tools has intensified [4].

Conventional field indicators, such as rigor mortis, hypostasis, and gross decomposition, are highly sensitive to temperature, humidity, and scavenging, limiting standardization across veterinary cases [1]. Organ-focused studies in animals, including experimental work in rabbits and pigs, suggest that tissue-specific trajectories can improve PMI inference, but precision and reproducibility remain limited under variable environmental histories [5–7]. Controlled-model pathology has further clarified post-mortem histological change, including rapid renal autolysis, highlighting the kidney as a promising target for PMI estimation [8, 9].

Molecular approaches aim to reduce subjectivity by quantifying degradation processes. Post-mortem RNA decay measured by reverse transcription quantitative polymerase chain reaction (RT-qPCR) has been proposed as a “molecular clock,” with evidence that RNA integrity and gene-specific cycle threshold (Ct) values correlate with PMI, although effects vary by tissue, gene, and conditions [2, 3, 10, 11]. In parallel, protein degradation signatures, captured by proteomics or targeted Western blotting, show time-dependent stability differences that can serve as PMI markers, including in renal tissue [12–15]. Evidence increasingly indicates that combining RNA and protein biomarkers improves PMI prediction compared with single-marker strategies [16–18].

Despite these advances, significant gaps remain in veterinary forensic literature. Most existing animal PMI studies rely on a single analytical modality, and very few have simultaneously integrated histopathological autolysis scoring, RNA degradation profiling, and protein degradation analysis from the same tissue samples within a single cohort [16, 19]. Furthermore, direct head-to-head comparisons between classical regression and machine-learning models for PMI prediction in a veterinary forensic context are scarce [19–21]. Analytically, advanced models such as random forests and support vector regression can exploit non-linear patterns in multimodal post-mortem datasets, potentially outperforming simple linear relations [19]. Without such integrated, organ-specific frameworks, PMI estimation in veterinary cases remains subjective and insufficiently validated, limiting its utility in legal, welfare, and biosecurity investigations [4].

The present study aimed to address these gaps by combining renal histopathology, RT-qPCR targeting heat shock protein 70 (*HSP70*), glyceraldehyde-3-phosphate dehydrogenase (*GAPDH)*, and *18S rRNA*, and Western blotting for *HSP70*, Caspase-3, and LC3 in Wistar rat kidneys across defined PMIs of 0, 6, 12, 24, and 48 h under controlled temperature conditions. These markers were selected for their complementary post-mortem kinetics: *HSP70* as a declining stress-response protein; *GAPDH* and *18S rRNA* as reference transcripts with documented renal decay profiles, Caspase-3 to capture early apoptotic signaling [22]; and LC3 to reflect autophagy-related dynamics in the intermediate PMI window [23]. Integrated datasets were entered into both linear regression and machine-learning models to develop an organ-specific, multimodal PMI estimation framework relevant to veterinary forensic practice [5, 6, 7, 9, 14, 15, 16, 19, 24]. Specifically, this study aimed to: (1) characterize time-dependent renal histopathological autolysis scores across a 0–48 h PMI window; (2) quantify post-mortem RNA decay trajectories for *HSP70*, *GAPDH*, and *18S rRNA* by RT-qPCR; (3) assess protein degradation kinetics of *HSP70*, Caspase-3, and LC3 by Western blotting; and (4) integrate these multimodal datasets into linear regression and machine-learning models to develop and compare organ-specific PMI prediction frameworks.

To our knowledge, this is the first study to simultaneously quantify renal histopathological autolysis scores, gene-specific RNA decay (*HSP70*, *GAPDH*, and *18S rRNA*), and protein degradation kinetics (*HSP70*, Caspase-3, LC3) within the same kidney samples across a 0–48 h PMI window in a controlled rat model. Most existing animal PMI studies rely on a single analytical modality, and very few have integrated histology, RNA, and protein data from matched tissue samples within a single cohort. Furthermore, direct head-to-head comparisons between classical linear regression and machine-learning models for PMI prediction in a veterinary forensic context remain scarce. Beyond its scientific contribution, this study benchmarks interpretable linear regression against machine-learning models on a matched multimodal dataset, clarifying the accuracy–interpretability trade-off relevant to veterinary forensic practice.

## MATERIALS AND METHODS

### Ethical approval

This study was conducted in accordance with internationally accepted principles for the ethical use of laboratory animals and complied with institutional guidelines governing animal care and use. Ethical approval was obtained from the Animal Ethics Committee of Lembaga Riset & Pengabdian Satwa Sehat Indonesia (LRP-SSI), Indonesia (Approval No. 016/EC-LRP-SSI/I/2025, dated January 1, 2025), before commencement of the study.

All experimental procedures adhered to the principles outlined in the Guide for the Care and Use of Laboratory Animals, the ARRIVE 2.0 guidelines, and the internationally recognized 3Rs principles (Replacement, Reduction, and Refinement). Adult male Wistar rats were housed under standardized environmental conditions with controlled temperature and humidity and a 12 h light/12 h dark photoperiod, and were provided *ad libitum* access to a standard laboratory diet and clean drinking water. Animals were acclimatized before the experiment and monitored daily by trained personnel to ensure their health and welfare throughout the study period. Veterinary oversight was maintained throughout the experimental phase, and any signs of pain, distress, or illness would have resulted in immediate veterinary intervention.

To minimize pain and distress, euthanasia was performed by trained personnel using an overdose of sodium pentobarbital (150 mg/kg body weight, intraperitoneally), resulting in rapid loss of consciousness followed by death in accordance with accepted veterinary euthanasia guidelines. Death was confirmed before tissue collection commenced. No procedures involving unnecessary pain, prolonged restraint, or repeated invasive interventions were performed. All subsequent investigations were conducted exclusively on post-mortem tissues.

The study was designed using the minimum number of animals required to achieve adequate statistical power while maintaining scientific validity. Histopathological, RNA, and protein analyses were performed on matched kidney samples collected from the same animals, thereby maximizing the scientific information obtained from each specimen while minimizing the total number of animals used. Carcasses were handled respectfully throughout the post-mortem investigation and were disposed of in accordance with institutional biosafety and biomedical waste management regulations. This ethical framework ensured compliance with high standards of animal welfare while generating scientifically robust and reproducible data for veterinary forensic research.

### Study period and location

All experimental procedures, including animal euthanasia, carcass storage, and sample collection, were performed between January and March 2025 under controlled laboratory conditions. The study was conducted at the Laboratory of Anatomic Pathology and Laboratory of Microbiology, Faculty of Veterinary Medicine, Universitas Brawijaya, Malang 65151, Indonesia.

### Study design

This controlled laboratory study used a rat model to characterize post-mortem renal change at histological, RNA, and protein levels, building on prior work on molecular PMI indicators and renal autolysis [9–11, 13, 15, 16]. Adult male Wistar rats were euthanized and carcasses maintained at approximately 25°C to approximate indoor veterinary forensic conditions. Kidneys were sampled at 0, 6, 12, 24, and 48 h post-mortem. For each time point, tissues were allocated to histopathology, RT-qPCR, and Western blotting. Histological scores, RNA measures, and protein profiles were integrated into regression and exploratory machine-learning models to estimate PMI [2, 3, 16, 19].

All data collection and analysis were completed. Full technical specifications including reagents, primer sequences, thermocycling conditions, and analysis code are provided below. Biomarkers were selected to capture complementary biological processes occurring during post-mortem tissue degradation. *HSP70* was included as a relatively stable stress-response marker, Caspase-3 as an indicator of apoptosis-related protein degradation during early PMI, and LC3 as a marker of autophagy-related cellular responses. The combination of these markers was intended to represent distinct degradation kinetics and improve PMI prediction performance when integrated with histopathological and RNA-based measurements.

### Animals and sampling

Adult male Wistar rats (200–250 g) were used to reduce biological variability, consistent with PMI research practice [11, 16]. Twenty-five animals were used (five per PMI time point, total n = 25). Animals were euthanized by intraperitoneal injection of sodium pentobarbital (150 mg/kg), and carcasses were subsequently stored intact at approximately 25°C. At each interval, kidneys were collected via midline incision and subdivided into: (i) formalin-fixed tissue for histopathology, (ii) snap-frozen cortex for RNA extraction, and (iii) aliquots for protein extraction. Ambient temperature, humidity, and elapsed time were recorded at the time of sampling. All histological, RNA, and protein analyses were performed on matched subsamples originating from the same kidney specimen collected from each animal. Sample size was determined based on previous PMI biomarker studies in rat models that reported detectable temporal molecular changes, with 4–6 biological replicates per time point. A priori power analysis (α = 0.05, power = 0.80) indicated that five animals per time point would be sufficient to detect large effect sizes (f ≥ 0.40) in repeated biomarker comparisons.

### Histopathology

Kidneys were fixed in 10% neutral-buffered formalin, paraffin-embedded, and stained with hematoxylin–eosin using standard protocols [8, 9]. Autolysis features were assessed (e.g., tubular epithelial disruption, nuclear fading/lysis, detachment, and glomerular disintegration) as described in animal models [8, 9, 15]. Two blinded observers scored tubular and glomerular autolysis on a 5-point ordinal scale; discrepancies >1 grade were resolved by consensus. Per-timepoint medians and interquartile ranges were calculated, and scores were used as predictors in PMI models [15, 16]. The 5-point ordinal scale defined each grade by distinct tubular and glomerular morphological features (Table 1).

**Table 1:** Five-point ordinal scale for renal autolysis scoring.

**Grade (score)**	**Tubular epithelial changes**	**Glomerular changes**
Grade 1 (minimal)	Intact epithelium; preserved nuclei and brush borders; occasional early chromatin margination	Architecture intact
Grade 2 (mild)	Scattered nuclear pyknosis; partial brush-border loss (<25% of tubules); minimal cytoplasmic vacuolation	Glomeruli intact
Grade 3 (moderate)	Widespread nuclear karyorrhexis/fading; brush-border loss 25–50%; early tubular epithelial detachment	Mild tuft retraction
Grade 4 (marked)	Extensive nuclear lysis; epithelial detachment (>50%); luminal cellular debris	Capillary disorganization
Grade 5 (severe)	Near-complete loss of tubular architecture with ghost outlines; confluent cellular debris	Glomerular disintegration

### RNA extraction and RT-qPCR

Renal cortex samples were snap-frozen and total RNA extracted using TRIzol reagent (Invitrogen, Thermo Fisher Scientific Inc., Waltham, MA, USA). RNA quantity and purity were assessed using a NanoDrop spectrophotometer (Thermo Fisher Scientific Inc., Waltham, MA, USA) prior to cDNA synthesis. RT-qPCR was performed on a QuantStudio 1 Real-Time PCR system (Applied Biosystems, Thermo Fisher Scientific Inc.) targeting *HSP70*, *GAPDH*, and *18S rRNA*, based on evidence of post-mortem stability [11, 16, 24]. Thermal cycling consisted of an initial denaturation at 94°C for 3 min, followed by 40 cycles of denaturation at 94°C for 30 sec, annealing at 56°C for 30 sec, and extension at 72°C for 25 sec. Primer sequences were adopted from previously validated studies and are listed in Table 2[25, 26]. Reactions were run in technical triplicate with no-template and no-RT controls [2, 3, 11, 24]. Outputs were expressed as ΔCt/ΔΔCt trajectories relative to 0 h. Melt-curve analysis was performed from 65–95°C with 0.5°C increments. A single peak was required to confirm amplification specificity.

**Table 2:** Primer sequences for reverse transcription quantitative polymerase chain reaction targets.

**Gene**	**Forward primer (5’-3’)**	**Reverse primer (5’-3’)**	**Amplicon length (bp)**	**Reference**
*HSP70*	ATGCTTCAGACCTCCCTT	CTCCACCAACTATCTCCACT	192	[25]
*GAPDH*	ACAGCAACAGGGTGGTGGAC	TTTGAGGGTGCAGCGAACTT	252	[25]
*18S rRNA*	GTAACCCGTTGAACCCCATT	CCATCCAATCGGTAGTAGCG	151	[26]

### Protein extraction and Western blot

Renal aliquots were homogenized in RIPA buffer with protease inhibitors. Equal protein amounts were separated by sodium dodecyl sulfate-polyacrylamide gel electrophoresis, transferred to polyvinylidene difluoride membranes, and detected by chemiluminescence following established post-mortem protocols [12, 14, 15]. Antibodies targeted *HSP70* (stress-response), Caspase-3 (apoptosis), and LC3 (autophagy) [12–16]. Primary antibodies were mouse monoclonal anti-*HSP70* (Santa Cruz Biotechnology, Dallas, TX, USA, Cat# sc-32239, 1:1000), rabbit polyclonal anti-Caspase-3 (Cell Signaling Technology, Danvers, MA, USA, Cat# 9662, 1:1000), rabbit polyclonal anti-LC3A/B (Cell Signaling Technology, Cat# 4108, 1:1000), and mouse monoclonal anti-GAPDH as a loading control (Santa Cruz Biotechnology, Cat# sc-32233, 1:2000). Bands were visualized using enhanced chemiluminescence (ECL) substrate (Thermo Fisher Scientific, Waltham, MA, USA) and imaged on a ChemiDoc system (Bio-Rad Laboratories, Hercules, CA, USA). Bands were quantified by standardized image analysis; normalized intensities and, where applicable, cleaved/pro-Caspase-3 ratios were computed for modeling [14, 16, 24]. Western blot densitometry and image-based quantification were performed using coded image files, and investigators were blinded to PMI groups until completion of data analysis.

### Integrated multimodal pipelines

Histopathological scores, RT-qPCR measurements, and Western blot-derived protein intensities were generated from matched kidney samples obtained from the same animal at each PMI interval. These datasets were subsequently integrated into a unified analytical framework linking morphological, transcriptional, and proteomic indicators of post-mortem degradation. The multimodal workflow was designed to evaluate whether combining complementary biological layers improved PMI estimation compared with single-modality approaches.

### Statistical analysis

Histopathology scores, Ct values, and normalized protein intensities were compiled into a database linked to animal ID and PMI, with checks for transcription errors, outliers, and distributional assumptions [2, 3, 24]. Group comparisons used one-way analysis of variance (ANOVA) (with post-hoc tests) for normally distributed variables and Kruskal–Wallis (with adjusted pairwise comparisons) otherwise; α = 0.05 (two-sided) [13, 24]. Correlation analyses assessed biomarker–PMI associations and inter-marker relationships [11, 16]. Normality was assessed using the Shapiro–Wilk test and homogeneity of variance using Levene’s test. For post-hoc analyses, Tukey’s HSD was applied following ANOVA and Dunn’s test with Benjamini–Hochberg adjustment following Kruskal–Wallis analysis. All statistical analyses were performed using R version 4.5.0 (R Foundation for Statistical Computing, Vienna, Austria; https://www.r-project.org/). Machine-learning analyses were implemented using the randomForest package (version 4.7-1.2; https://cran.r-project.org/package=randomForest) and e1071 package (version 1.7-17; https://cran.r-project.org/package=e1071). Western blot densitometry was performed using Fiji ImageJ version 1.54p (National Institutes of Health, Bethesda, MD, USA; https://imagej.net/software/fiji/).

**Predictive modeling of PMI:** Multiple linear regression models were built with PMI as the outcome and histology, RNA, and protein variables as predictors [16, 19]. random forest and support vector machine regressors were also implemented to capture non-linear relationships [19]. The random forest model used 500 trees; the support vector regression used a radial basis function kernel with cost = 10 and gamma set by the scale heuristic. Given the small sample size (n = 25), leave-one-out cross-validation was used as the primary scheme, with 5-fold cross-validation repeated 10 times as a sensitivity check; both gave concordant model rankings. Performance was summarized by mean absolute error (MAE), root mean square error (RMSE), and R². A secondary analysis used discretized PMI classes to summarize classification accuracy. Feature importance (random forest) and reduced-predictor sensitivity analyses were used to identify robust predictors [16, 19].

**Quality assurance and reproducibility:** Histology scoring was blinded; sample processing was randomized across time points and assay runs to reduce batch effects. The raw Ct tables, processed datasets, metadata, and analysis scripts are available from the corresponding author on reasonable request.

## RESULTS

### Implementation of the research plan

All planned study stages were completed using adult male Wistar rats (~250 g). The full workflow, from euthanasia to sampling and laboratory analyses, was executed at fixed PMIs of 0, 6, 12, 24, and 48 h, with carcasses maintained at 25°C to standardize environmental conditions and treat PMI as a discrete time variable. At each interval, both kidneys were collected via midline abdominal incision and partitioned to generate matched samples for histopathology, RNA, and protein analyses. No major procedural deviations occurred, and all intended measurements were obtained. Histopathology samples underwent routine fixation, processing, and hematoxylin–eosin staining for semiquantitative autolysis scoring; RNA samples were snap-frozen and analyzed by RT-qPCR for *HSP70*, *GAPDH*, and *18S rRNA*; and protein aliquots were processed for Western blotting of *HSP70*, Caspase-3, and LC3. This workflow generated a complete dataset of histological scores, Ct values, and normalized protein measures across all PMIs.

### Histopathology, RNA, and protein trajectories across PMI

**Figure 1:**
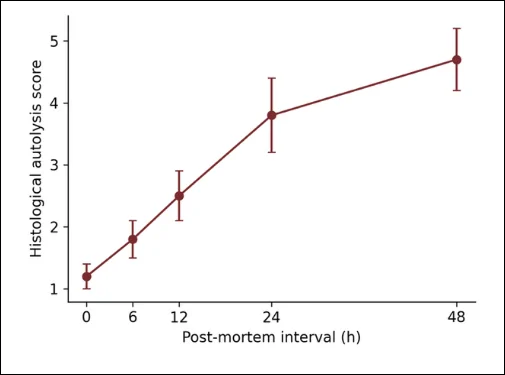
Progressive increase in renal autolysis scores during the post-mortem interval.

**Figure 2:**
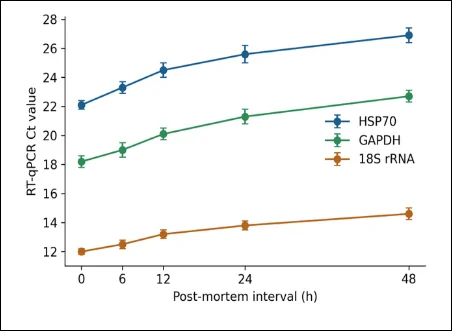
Temporal increase in reverse transcription quantitative polymerase chain reaction cycle threshold (Ct) values of renal RNA markers during the post-mortem interval. Ct values increased progressively for all targets, indicating ongoing RNA degradation. Error bars represent standard deviation (mean ± SD).

**Figure 3:**
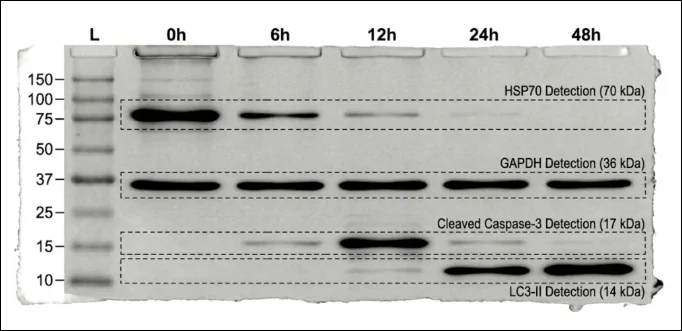
Western blot analysis of renal protein changes across the post-mortem interval. Representative immunoblots at 0, 6, 12, 24, and 48 h post-mortem (25°C): (A) *HSP70* (~70 kDa) declined progressively; (B) GAPDH (~36 kDa), loading control, remained stable across all lanes; (C) cleaved Caspase-3 (~17 kDa) peaked at 12 h; (D) LC3-II (~14 kDa) increased and plateaued by 24–48 h. The full, uncropped membrane with molecular-weight markers (kDa) is shown at left; quantification was based on n = 5 per time point. GAPDH = Glyceraldehyde-3-phosphate dehydrogenase.

**Figure 4:**
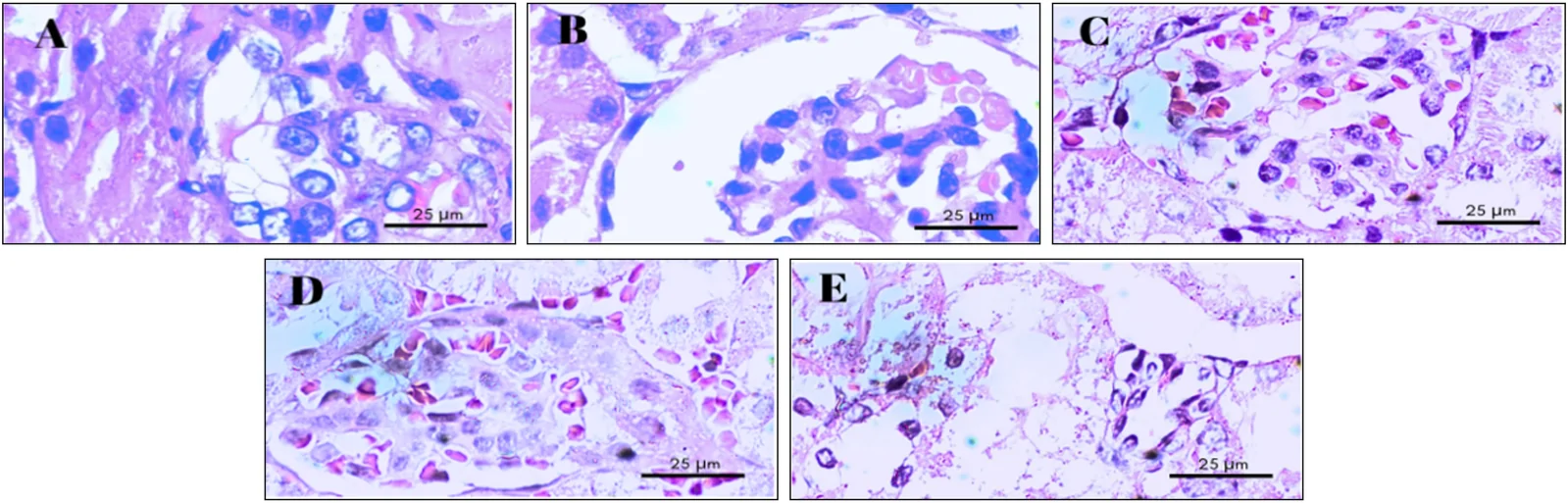
Representative renal histopathology (H&E) across the post-mortem interval. Renal cortex at (A) 0 h, (B) 6 h, (C) 12 h, (D) 24 h, and (E) 48 h post-mortem (25°C). Progressive autolysis is evident—from intact tubular epithelium with well-preserved basophilic nuclei and intact glomeruli at 0–6 h, through nuclear pyknosis/karyorrhexis, brush-border loss and early epithelial detachment at 12–24 h, to near-complete loss of tubular architecture with ghost outlines, eosinophilic debris and glomerular disintegration at 48 h. These changes parallel the ordinal autolysis scores (mean 1.2 → 4.7; Table 3). Scale bar = 25 µm, applies to all panels (same magnification); n = 5 per time point.

Core quantitative outcomes are summarized in Table 3. Renal autolysis scores rose progressively from 0 to 48 h, increasing from 1.2 ± 0.2 at 0 h to 4.7 ± 0.5 at 48 h, with the steepest increases occurring between 12–24 h and 24–48 h (Figure 1). RT-qPCR Ct values increased monotonically for all targets, consistent with progressive transcript loss: *HSP70* increased from 22.1 ± 0.3 to 26.9 ± 0.5; *GAPDH* from 18.2 ± 0.4 to 22.7 ± 0.4; and *18S rRNA* from 12.0 ± 0.2 to 14.6 ± 0.4 (Figure 2). Protein measures showed marker-specific dynamics (Figure 3). Normalized HSP70 protein declined continuously from 1.00 ± 0.05 to 0.35 ± 0.05. Caspase-3 increased from 0.25 ± 0.04 to a peak at 12 h (1.00 ± 0.06) and then decreased to 0.40 ± 0.05 by 48 h. LC3-II increased from 0.25 ± 0.03 to 1.15 ± 0.03, rising markedly up to 12–24 h and approaching a plateau thereafter. Representative renal histopathology (H&E) at each interval is shown in Figure 4.

### Statistical associations and PMI prediction performance

**Figure 5:**
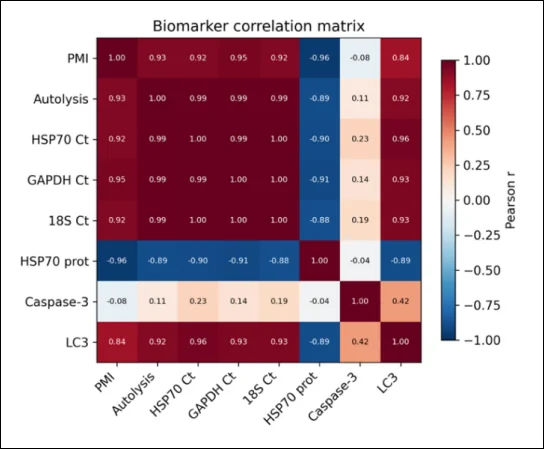
Correlation matrix of histological, RNA and protein biomarkers with post-mortem interval (Pearson r). Autolysis score, RNA Ct values and LC3-II correlated positively with PMI, HSP70 protein correlated negatively, and Caspase-3 showed no significant linear correlation (consistent with its non-monotonic 12 h peak). n = 25.

**Correlation analysis (n = 25) showed strong associations between PMI and most markers:** autolysis score (Pearson r = +0.93), *HSP70* Ct (+0.92), *GAPDH* Ct (+0.95), *18S rRNA* Ct (+0.92), LC3-II (+0.84), and HSP70 protein (r = −0.96; all p < 0.001). Caspase-3 showed no significant linear correlation with PMI (r = −0.08; p = 0.70), consistent with its non-monotonic peak at 12 h, but contributed temporal structure when combined with other markers. The full biomarker correlation matrix is shown in Figure 5.

**Table 3:** Histopathological score, RNA cycle threshold (Ct) values, and protein expression levels in rat renal tissue at different post-mortem intervals (mean ± SD).

**Post-mortem interval (h)**	**Histological autolysis score**	***HSP70*** ** Ct**	***GAPDH*** ** Ct**	***18S rRNA*** ** Ct**	**HSP70** ** protein**	**Caspase-3 protein**	**LC3-II**
0	1.2 ± 0.2^d^	22.1 ± 0.3^e^	18.2 ± 0.4^d^	12.0 ± 0.2^d^	1.00 ± 0.05^a^	0.25 ± 0.04^d^	0.25 ± 0.03^d^
6	1.8 ± 0.3^c^	23.3 ± 0.4^d^	19.0 ± 0.5^d^	12.5 ± 0.3^d^	0.90 ± 0.06^a^	0.60 ± 0.05^b^	0.60 ± 0.04^c^
12	2.5 ± 0.4^c^	24.5 ± 0.5^c^	20.1 ± 0.4^c^	13.2 ± 0.3^c^	0.75 ± 0.04^b^	1.00 ± 0.06^a^	0.90 ± 0.05^b^
24	3.8 ± 0.6^b^	25.6 ± 0.6^b^	21.3 ± 0.5^b^	13.8 ± 0.3^b^	0.55 ± 0.06^c^	0.65 ± 0.05^b^	1.10 ± 0.04^a^
48	4.7 ± 0.5^a^	26.9 ± 0.5^a^	22.7 ± 0.4^a^	14.6 ± 0.4^a^	0.35 ± 0.05^d^	0.40 ± 0.05^c^	1.15 ± 0.03^a^

Values are mean ± SD (n = 5 per time point). For each parameter, one-way ANOVA was significant (all p < 0.0001; df = 4, 20): autolysis F = 55.8; *HSP70* Ct F = 79.1; *GAPDH* Ct F = 86.2; *18S rRNA* Ct F = 53.6; HSP70 protein F = 121.9; Caspase-3 F = 162.2; LC3-II F = 461.6. Within each column, means not sharing a common superscript letter differ significantly (Tukey's HSD, p < 0.05); letter "a" denotes the highest mean. Autolysis score, as an ordinal variable, was additionally confirmed by Kruskal–Wallis (H = 22.2; p < 0.001). GAPDH = Glyceraldehyde-3-phosphate dehydrogenase.

A multiple linear regression model was constructed using PMI (hours) as the outcome and histological score, LC3-II, Caspase-3, and HSP70 protein as predictors: PMI (h) = 57.77 + 9.16 × (histological score) − 31.10 × (LC3-II) + 4.68 × (Caspase-3) − 60.90 × (HSP70 protein).

Model benchmarking compared multiple linear regression, random forest regression, and support vector regression (radial basis kernel). Performance metrics are shown in Table 4. Across models, R2 ranged from 0.95 to 0.99, MAE from 0.75 to 2.83 h, and accuracy within +/-3 h from 56% to 92%. Support vector regression achieved the lowest MAE (0.75 h) and highest +/-3 h accuracy (92%), followed by random forest (MAE 1.54 h; 84%), whereas multiple linear regression performed worst (MAE 2.83 h; 56%). 

Regression coefficients and uncertainty. For the multiple linear regression model, the unstandardized coefficients (B), standard errors (SE), 95% confidence intervals, and p-values were: histological score, B = 9.16 (SE 2.31; 95% confidence interval [CI] 4.33–13.98; p = 0.001); LC3, B = −31.10 (SE 16.98; 95% CI −66.52 to 4.32; p = 0.082); Caspase-3, B = 4.68 (SE 8.31; 95% CI −12.65 to 22.01; p = 0.579); HSP70 protein, B = −60.90 (SE 12.07; 95% CI −86.08 to −35.72; p < 0.001); and intercept, B = 57.77 (SE 12.68; p < 0.001). The overall fit was high in-sample (R² = 0.97; adjusted R² = 0.97; F(4, 20) = 173.0; p < 0.001), but variance inflation factors were large (histological score 23.8; LC3 81.4; Caspase-3 11.4; HSP70 protein 20.8; all well above the conventional threshold of 5–10), indicating substantial multicollinearity. Consequently, only histological score and HSP70 protein reached significance; the negative LC3 coefficient is counterintuitive and unstable, and the model is best regarded as predictive rather than explanatory.

**Figure 6:**
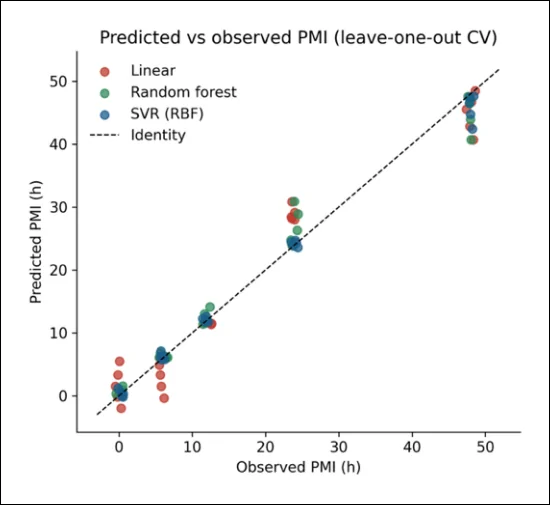
Predicted versus observed post-mortem interval under leave-one-out cross-validation for multiple linear regression, random forest and support vector regression (Radial basis function). The dashed line is the identity. Support vector regression showed the closest agreement (MAE 0.75 h); linear regression showed the widest scatter, consistent with multicollinearity-related instability. n = 25.

Machine-learning interpretability and classification. In the random forest model, permutation importance ranked HSP70 protein as the dominant predictor (0.78), followed by LC3-II (0.13), histological score (0.05), and Caspase-3 (0.02). When PMI was discretized into five ordinal classes (0, 6, 12, 24, and 48 h), random forest classification under leave-one-out cross-validation reached 100% accuracy (Cohen’s κ = 1.00); this near-perfect separation reflects the controlled experimental design and well-spaced sampling intervals rather than field-realistic conditions. For continuous PMI prediction, support vector regression achieved the lowest error (MAE: 0.75 h), outperforming random forest (MAE: 1.54 h) and linear regression (MAE: 2.83 h) (Table 4). This accuracy is competitive with recent animal and multi-tissue molecular PMI models [2, 3, 20]. Cross-validated predicted versus observed PMI for all three models is shown in Figure 6.

**Table 4:** Performance of predictive models for post-mortem interval estimation (leave-one-out cross-validation; n = 25).

**Model**	**MAE (hours)**	**RMSE (hours)**	**R²**	**Accuracy within ±3 h**
Linear regression	2.83	3.65	0.95	56%
Random forest	1.54	2.54	0.98	84%
Support vector regression (radial basis function) kernel	0.75	1.40	0.99	92%

## DISCUSSION

### Interpretation of renal autolysis scoring as a PMI marker

The monotonic increase in renal autolysis scores from 0–48 h aligns with evidence from controlled models that the kidney undergoes rapid and progressive post-mortem structural breakdown [8, 9]. The accelerations between 12–24 h and 24–48 h are consistent with a transition from early cellular changes to more advanced tissue disintegration, supporting the kidney as a sensitive substrate for PMI evaluation [9, 15]. Although histology alone is unlikely to deliver narrow PMI estimates across heterogeneous field conditions, its strong time-linked gradient and contribution to multivariable models indicate that autolysis scoring remains useful when integrated with molecular measures [16].

### RNA decay profiles and implications for molecular PMI inference

Ct values for *HSP70*, *GAPDH*, and *18S rRNA* increased steadily with PMI, consistent with progressive RNA degradation and reduced template availability. This pattern accords with reviews showing that gene-specific Ct trajectories often correlate with PMI, while remaining sensitive to tissue type and ambient conditions [2, 3, 10]. The present findings support the feasibility of kidney RNA markers for PMI estimation under controlled indoor temperature and reinforce recommendations to combine multiple transcripts with differing stability to enhance robustness [3, 11]. The near-linear spacing across intervals further suggests that, at 25°C, renal RNA decay may be sufficiently regular for regression-based prediction in early-to-intermediate PMI windows.

### Protein-level temporal structure and complementary marker behavior

**Protein results demonstrated distinct, complementary kinetics:** *HSP70* decreased monotonically, Caspase-3 showed an early peak, and LC3 increased and plateaued. These patterns fit established concepts that proteins differ in post-mortem stability and that apoptosis- and autophagy-related markers can show time-dependent shifts during early autolysis [12, 13, 14]. The Caspase-3 peak at 12 h likely reflects early post-mortem apoptotic signaling or proteolytic processing before later degradation, which may explain its weaker linear association with PMI when considered alone but its potential utility in multivariate models [14]. LC3 dynamics provided additional information concentrated in the early-to-intermediate window, supporting the rationale for multi-protein panels rather than single targets [15, 16].

**Modeling choices:** linear regression versus machine learning

Across cross-validated comparisons, machine-learning models outperformed multiple linear regression: support vector regression achieved the lowest MAE (0.75 h), followed by random forest (1.54 h), while linear regression performed worst (2.83 h). The poor generalization of the linear model is attributable to severe multicollinearity among the multi-level predictors (variance inflation factors up to 81), which inflated coefficient variance and produced an unstable in-sample fit (R² = 0.97) that did not transfer to held-out data. These results indicate that, for this matched multimodal panel, non-linear algorithms better capture the relationship between biomarker degradation and PMI. Linear regression nonetheless retains practical value when transparency and a closed-form equation take precedence over accuracy, provided multicollinearity is mitigated through predictor selection or regularization. Machine learning is likely to offer further advantage as datasets expand and as variable field conditions introduce non-linearities and interactions [16, 19].

### Implications, limitations, and future directions

These findings reinforce the kidney as a strong candidate organ for PMI estimation and show that combining histological scoring with RNA and protein profiles yields a richer temporal signal than morphology alone [1, 4]. However, generalizability is constrained by the single species, a controlled temperature (25°C), and the 0–48 h window. Field conditions, variable climates, carcass sizes, and species differences are likely to alter degradation kinetics and may reduce model transferability without recalibration [1]. The biomarker panel, while literature-grounded, may omit additional candidates with superior discrimination, and the modest sample size may limit the full utility of complex algorithms [12, 19]. Future studies should replicate across temperatures and humidity, extend to other species and organs, and evaluate multimodal integration with physicochemical and entomological indicators to build more robust, field-relevant PMI frameworks [2, 3, 5–7]. A further limitation is that RNA integrity was assessed only spectrophotometrically (A260/280) rather than by RNA integrity number (RIN)-based electrophoresis. While the progressive rise in Ct values is consistent with RNA degradation, direct RIN quantification would provide a more robust measure of integrity and is recommended for future studies.

## CONCLUSION

This study demonstrates that integrating renal histopathological autolysis scoring, RNA decay profiles (*HSP70*, *GAPDH*, and *18S rRNA*), and protein degradation kinetics (*HSP70*, Caspase-3, and LC3) from matched kidney samples in a Wistar rat model significantly enhances PMI prediction under controlled conditions. Key results showed progressive increases in autolysis scores (1.2 ± 0.2 at 0 h to 4.7 ± 0.5 at 48 h), monotonic rises in RT-qPCR Ct values across all RNA targets, and marker-specific protein dynamics (monotonic decline of *HSP70*, early peak of Caspase-3 at 12 h, and plateau of LC3-II after 24 h). Among the models evaluated, support vector regression achieved the highest accuracy (MAE 0.75 h, RMSE 1.40 h, R² 0.99, 92% within ±3 h), outperforming random forest and multiple linear regression.

The multimodal framework provides a scalable template for molecular veterinary forensic science. It can support more objective and precise PMI estimation in legal, welfare, biosecurity, and insurance cases involving animal deaths, particularly in indoor or controlled environments. The approach may also guide the development of field-applicable toolkits that combine rapid histological scoring with targeted molecular assays.

Strengths include the use of matched samples from the same kidney for all modalities, a well-controlled experimental design with multiple time points, direct comparison of classical regression and machine-learning algorithms, and comprehensive blinded analyses. This is the first veterinary study to benchmark these modeling approaches on an integrated renal histopathological, RNA, and protein dataset.

Limitations include the single-species (rat) model, controlled temperature (25°C), and a relatively short 0–48 h PMI window. Field conditions with variable temperature, humidity, carcass size, and scavenging were not evaluated. RNA integrity was assessed spectrophotometrically rather than by RIN, and while the sample size was powered for the primary outcomes, it was modest for complex machine-learning models.

Future studies should validate the framework across different species, temperatures, humidity levels, and longer PMI windows. Incorporation of additional omics layers, physicochemical indicators, and entomological data, along with larger independent validation cohorts and field-derived samples, will further strengthen model robustness and generalizability for real-world veterinary forensic applications.

In conclusion, combining renal histopathology, RNA decay, and protein degradation signatures with machine-learning models, particularly support vector regression, offers a promising, high-accuracy approach for PMI estimation in veterinary forensics. While further validation under diverse conditions is essential, these findings establish a solid methodological foundation that can advance precision and reliability in veterinary forensic investigations, ultimately supporting better outcomes in legal, welfare, and biosecurity contexts.

## DATA AVAILABILITY

All data supporting the findings of this study are available from the corresponding author on reasonable request.

## GENERATIVE AI DECLARATION

The authors declare that generative artificial intelligence (AI) tools were used solely to improve language, grammar, and readability during manuscript preparation. All scientific content, data analysis, interpretation of results, and conclusions were developed and verified by the authors. The authors take full responsibility for the accuracy, integrity, and originality of the work presented, and no AI tool was listed as an author.

## AUTHORS’ CONTRIBUTIONS

OPC, AH, and FNAEPD: Conceptualization and study design. OPC and AH: Investigation and data collection. OPC, AH, and FNAEPD: Data analysis and interpretation. OPC and AH: Drafted and revised the manuscript. All authors have read and approved the final version of the manuscript.
